# ATTITUDES TOWARD MOTIVATIONAL MESSAGING IN A DIGITAL HEALTH ACTIVITY PROGRAM FOR OLDER FAMILY CARE PARTNERS

**DOI:** 10.1093/geroni/igae098.2894

**Published:** 2024-12-31

**Authors:** Dawon Baik, Paul Cook, Catherine Jankowski

**Affiliations:** University of Colorado, Aurora, Colorado, United States; University of Colorado, Aurora, Colorado, United States; University of Colorado, Aurora, Colorado, United States

## Abstract

Older family care partners of patients with heart failure (HF-FCPs) often experience physical, psychological, and social health issues due to age-related health needs, less emotional support, and social isolation. We developed a tailored digital health physical activity (PA) program, TPA4You that includes videoconferencing with a health coach, wearable sensor, and motivational messaging. To refine TPA4You, a 1-week, two-phase, iterative field test of TPA4You was conducted. Herein, we report on older HF-FCPs’ perceptions and attitudes toward the motivational messaging. We used an existing motivational messaging system. Prior to generating messages, the system queried PA barriers. The system automatedly generated a message to overcome the barrier rated highest by the participant at the time of the survey. The seven participants in Phase 1 were women aged 68.3 ±7.1 yrs. (M±SD), predominantly non-Hispanic White (86%), and caring for a spouse (71%). Some attitudes toward the motivational messages were favorable, such as encouraged PA, provided logical and applicable advice, and reinforced a committed to exercise, while others were unfavorable, such as the messages were easily ignored, ineffective for increasing PA, and not sufficiently tailored to the perceived barrier. The messaging system was perceived as easy to use and quick to complete by some, while others felt that answering questions within the system was confusing, questions were repetitive, and response options too limited. Many reported twice weekly messaging was sufficient. These data will be used to modify the frequency, content, and system (automated vs. manual) of motivational messaging in Phase 2 of TPA4You modifications.

